# Association between Body Mass Index and Health-Related Quality of Life: The "Obesity Paradox" in 21,218 Adults of the Chinese General Population

**DOI:** 10.1371/journal.pone.0130613

**Published:** 2015-06-18

**Authors:** Yanbo Zhu, Qi Wang, Guoming Pang, Lin Lin, Hideki Origasa, Yangyang Wang, Jie Di, Mai Shi, Chunpok Fan, Huimei Shi

**Affiliations:** 1 School of Management, Beijing University of Chinese Medicine, Beijing, China; 2 School of Preclinical Medicine, Beijing University of Chinese Medicine, Beijing, China; 3 Internal Medicine Department, Kaifeng Hospital of Traditional Chinese Medicine, Kaifeng, Henan, China; 4 Department of Quality Management, China-Japan Friendship Hospital, Beijing, China; 5 Division of Biostatistics and Clinical Epidemiology, School of Medicine, University of Toyama, Toyama, Japan; 6 Department of Clinical Nutrition, China-Japan Friendship Hospital, Beijing, China; University of Catanzaro Magna Graecia, ITALY

## Abstract

**Background:**

There was no consistent recognition of the association between high or low body mass index (BMI) and health related quality of life (HRQL). The aim of this research was to study the association between BMI and HRQL in Chinese adults, and to further explore the stability of that association in the subgroup analysis stratified by status of chronic conditions.

**Methods:**

A total of 21,218 adults aged 18 and older were classified as underweight, normal weight, overweight, class I obese, and class II obese based on their BMI. HRQL was measured by the SF-36 Health Survey. The independent impact of each BMI category on HRQL was examined through standard least squares regression by comparing the difference of SF-36 scores and the minimum clinically important differences (MCID), which was defined as 3 points.

**Results:**

Compared to the normal weight, the class I obese was significantly associated with better HRQL scores in the mental component summary (MCS) (75.1 vs. 73.4, P<0.001). The underweight had the lowest score in both the physical components summary (PCS) (75.4 vs. 77.5, P<0.001) and mental components summary (MCS) (71.8 vs. 73.4, P<0.001). For the MCID, the HRQL score was reduced by more than 3 points in the physical functioning for the class II obese (D=-3.43) and the general health for the underweight (D=-3.71). Stratified analyses showed a similar result in the health subjects and chronic conditions, and it was significant in the chronic conditions.

**Conclusions:**

The class I obese showed the best HRQL, especially in the mental domain. The worst HRQL was found in the underweight. The class II obese reduced HRQL in the physical functioning only. “Obesity paradox” was more obvious in the participants with chronic conditions.

## Background

Overweight and obesity have reached epidemic proportions throughout the world [1–4]. In China, 21.8 percent of adults are overweight or obese [5]. As a major contributor to a variety of chronic diseases [6–9], obesity has become one of the important global public health issues.

The association between obesity and a poorer health-related quality of life (HRQL) has been exploredpreviously. Kortt [10] conducted a study to investigate the correlation between HRQL and BMI in a representative sample of the Australian’s general population and found that BMI was negatively associated with the SF-6D questionnaire score. Another research conducted by Jia [11] showed that HRQL decreased with increasing levels of obesity. Compared with normal weight respondents, persons with severe obesity had significantly lower scores on the PCS-12, MCS-12, EQ-5D index, and EQ VAS, at 4.0, 1.1, 0.073, and 4.8 points lower, respectively. Persons with moderate obesity or who are overweight also had significantly poorer HRQL. McDonough et al. [12] provided further evidence for the association between increasing BMI and poorer HRQL in a mixed population of white European (WE) and South Asian (SA) ethnicity, whilst suggested that SA ethnicity modify this relationship. Oreopoulos [13] reported that BMI was inversely associated with the physical function and overall HRQL in coronary artery disease (CAD) patients, especially those with severe obesity.

However, some data hasaccumulated suggesting survival benefit or improved HRQL in elderly or patients with existing chronic diseases who are overweight and moderately obese[14–19], especially in the mental domain quality of life [20–24]. Some research presented underweight associated with poorer HRQL[25,26]. We had previously proved that, to the middle-aged and the old, higher BMI was associated with better mental HRQL, while the physical and mental components of the HRQL were poorer in the population that was underweight [27].

There was no consistent recognition of the association between high or low body mass index and health related quality of life. Thus, the objective of this research was 1) to study the association between BMI and HRQL in the general adult population in Chinese after adjusting for potential confounders, and 2) to further explore the stability of that association between BMI and HRQL subgroup by status of chronic conditions.

## Methods

### Data Sources

Data collected from a cross-sectional survey of 21,948 casesover-15s in China from December 2005 to January 2007, of nine provinces and municipalities (East: Beijing, Jiangsu, and Fujian; Central: Anhui, Jiangxi, Jilin, and Henan; West: Gansu and Qinghai) [27]. The subjects were selected by tri-phase stratified sampling. Firstly, the 9 provinces and municipalities of China were randomly selected by the SAS program. Secondly, communities, colleges and health examination centers were selected by convenience sampling. Thirdly, community residents, students and subjects were drawn out from each community, college and health examination center by systemic sampling. Quality control of the program was incorporated into the design, implementation and the data processing under the organization and supervision of the Beijing University of Chinese Medicine. All the study subjects signed a written informed consent. It was supported by the National Program on Key Basic Research Project of China (973 Program) in 2005, which was organized by the Ministry of Science and Technology of the People’s Republic of China.

Information of participants was collected by responding to standard questionnaire including gender, age, marital status, educational level, exercise habit, use of tobacco and alcohol, districts of residence, chronic conditions, weight, height, and HRQL. The chronic diseases included hypertension, hyperlipidemia, diabetes, cerebral apoplexy, cardiopathy (myocardial infarction, coronary artery disease), hepatopathy (fatty liver, alcoholic hepatitis, hepatocirrhosis), gastric ulcer, Cancer, osteoporosis, bronchial asthma, which were collected from the self-reported results by respondents.110 individuals (0.5 percent) with missing values distributed in the value of educational level, use of tobacco and alcohol, exercise habit and chronic conditions. The imputation model was supplied to handle the missing values, and 21,218 cases were included in this study after excluding persons under 18 years of age.

### Measurement of HRQL

HRQL was measured using Chinese version 1.0 of the SF-36 Health Survey [28] developed in 1997. The SF-36 [29,30] is made up of 36 questions that correspond to eight dimensions, including physical functioning (PF), role physical (RP), bodily pain (BP), general health (GH), vitality (VT), social functioning (SF), and role emotional(RE) and mental health (MH). All dimensions can be further classified into the physical domain (PF, RP, BP, and GH) and the mental domain (VT, SF, RE and MH). The score scale for each category assigns scores from 0 to 100, with 100 representing the best health status while0 representing the worst. The SF-36 scores can also be expressed as two summary measures, the physical component summary (PCS) and the mental component summary (MCS), which provides a measure of the overall effect of physical and mental impairment.

### Categorization of BMI

In this study, the data of weight and height was self-reported by the participants. Body weight was classified by WHO guidelines for the Asian Pacific population(WHO/IASO/IOTF, 2000) [31] into five BMI (kg/m^2^)categories: underweight (<18.5), normal weight (18.5–22.9), overweight (23–24.9),class I obese (25–29.9) and class II obese (≥30.0).

### Analysis of the Data

We have previously proved that age, gender, marital status, educational level, exercise habit, BMI and chronic diseases were the main predictive factors of HRQL in general population of China[32]. The use of tobacco [33,34], alcohol [35,36] or socio-economic condition [37,38] were associated factors on HRQL. Therefore, standard least squares regression (adjusted for gender, age, marital status, educational level, exercise habit, use of tobacco and alcohol, districts of residence, chronic conditions and BMI-chronic condition interaction) was used to estimate the independent impact of each BMI category on overall HRQL. We also verified that chronic disease was one of the first two predictive factors in HRQL [32]. Thus, the stratified model was run subgroup by chronic conditions to further explore the stability of that association.

Some studies have suggested that a difference of 3 to 5 points may be regarded as a minimum clinically important difference (MCID) [39,40]. In this study, we used Bonferonni correction for multiple comparisons and interpreted the results based on statistical significance (P<0.05* (1/4) = 0.0125≈0.01) as well as MCID defined as 3 points. All the statistical analyses were performed using JMP 10.0 (SAS Institute Inc.).

## Results

### Characteristics of the Subjects

The mean age of the 21,218 participants was 35.4 (SD 14.7) years, ranged from 18 to 92 years, 46.8% were male, 58.2% were married, 55.4%were college or above education level, and 27.5% had at least one chronic disease. Categorized by BMI, the proportions of the subjects were 11.4%, 55.8%, 17.1%, 14.5%, and 1.3% for the underweight, normal weight, overweight, class I obese and class II obese. The characteristics of the participants did not equally distribute among the BMI categories (P<0.001). For example, higher proportions of overweight and obesity were found in older males and people who had primary or below educational level, use of tobacco and alcohol, and one chronic disease. (Table 1).

**Table 1:** Characteristics of subjects by BMI categories.

Characteristics	Overall (*n* = 21218)	Underweight (*n* = 2413)	Normal weight (*n* = 11836)	Overweight (*n* = 3621)	Class I obese (*n* = 3078)	Class II obese (*n* = 270)
**Gender**						
Men	9930	682(6.87)	5126(51.62)	2018(20.33)	1947(19.61)	156(1.57)
Women	11288	1731(15.33)	6710(59.44)	1602(14.19)	1131(10.02)	114(1.01)
**Age (years)**						
18–44	15896	2182(13.73)	9596(60.37)	2316(14.57)	1653(10.40)	149(0.94)
45–59	3542	127(3.59)	1506(42.52)	878(24.79)	944(26.65)	87(2.46)
60–92	1780	104(5.84)	734(41.24)	427(23.99)	481(27.02)	34(1.91)
**Age ($\bar{x}$±s)**	21218	27.8±12.3	33.0±13.8	40.5±14.3	44.2±14.0	43.7±13.9
**Marital status**						
Unmarried	8273	1591(19.23)	5415(65.45)	801(9.68)	432(5.22)	34(0.41)
Married	12352	784(6.35)	6125(49.59)	2701(21.87)	2519(20.39)	223(1.81)
Other	593	38(6.41)	296(49.92)	119(20.07)	127(21.42)	13(2.19)
**Educational level**						
Primary or below	1311	87(6.64)	665(50.72)	263(20.06)	260(19.83)	36(2.75)
Junior high and high school	8152	829(10.17)	4336(53.19)	1540(18.89)	1333(16.35)	114(1.40)
College or above	11755	1497(12.74)	6835(58.15)	1818(15.47)	1485(12.63)	120(1.02)
**Exercise habit**						
Often	4618	374(8.10)	2568(55.61)	867(18.77)	750(16.24)	59(1.28)
Sometimes	9258	1081(11.68)	5288(57.12)	1538(16.61)	1259(13.60)	92(0.99)
Less	7342	958(13.05)	3980(54.21)	1216(16.56)	1069(14.56)	119(1.62)
**Use of Tobacco**						
No	18357	2238(12.19)	10493(57.16)	3022(16.46)	2394(13.04)	210(1.14)
Yes	2861	175(6.12)	1343(46.94)	599(20.94)	684(23.91)	60(2.10)
**Use of Alcohol**						
No	18778	2302(12.26)	10751(57.25)	3083(16.42)	2449(13.04)	193(1.03)
Yes	2440	111(4.55)	1085(44.47)	538(22.05)	629(25.78)	77(3.16)
**Chronic conditions**						
No	15385	1980 (12.87)	9327(60.62)	2446(15.90)	1543(10.03)	89(0.58)
Yes*	5833	433(7.42)	2509(43.01)	1175(20.14)	1535(26.32)	181(3.10)
Hypertension						
*No*	19752	2380(12.05)	11428(57.86)	3273(16.57)	2475(12.53)	196(0.99)
*Yes*	1466	33(2.25)	408(27.82)	348(23.74)	603(41.13)	74(5.05)
Hyperlipidemia						
*No*	20305	2404(11.84)	11618(57.22)	3417(16.83)	2656(13.08)	210(1.03)
*Yes*	913	9(0.99)	218(23.88)	204(22.34)	422(46.22)	60(6.57)
Diabetes						
*No*	20806	2404(11.55)	11678(56.13)	3512(16.88)	2960(14.23)	252(1.21)
*Yes*	412	9(2.18)	158(38.35)	109(26.46)	118(28.64)	18(4.37)
Heart disease						
*No*	20568	2388(11.61)	11601(56.40)	3453(16.79)	2878(13.99)	248(1.21)
*Yes*	650	25(3.85)	235(36.15)	168(25.85)	200(30.77)	22(3.38)
Other**						
*No*	17879	2043(11.43)	10114(56.57)	3043(17.02)	2473(13.83)	206(1.15)
*Yes*	3339	370(11.08)	1722(51.57)	578(17.31)	605(18.12)	64(1.92)
**Districts**						
East	8368	1013(12.11)	4622(55.23)	1391(16.62)	1233(14.73)	109(1.30)
Central	8342	840(10.07)	4586(54.97)	1525(18.28)	1273(15.26)	118(1.41)
West	4508	560(12.42)	2628(58.30)	705(15.64)	572(12.69)	43(0.95)

(1) Overall distributions among four BMI categories were significantly different with p<0.001. (2) BMI (kg/m^2^) categories: Underweight (<18.5), Normal weight (18.5–22.9), Overweight (23–24.9), Class I obese (25–29.9), Class II obese (≥30.0). (3)

*Because affected individuals may suffer from more than one chronic disease, so the sum of the total Yes of hypertension, hyperlipidemia, diabetes, heart disease and other is greater than 5833 cases.

**Including gastric ulcer, cancer, cerebral stroke, bronchial asthma, osteoporosis, etc.

### Correlation between BMI and HRQL

Overall, the results showed the mean scores of HRQL increased with increasing body mass from the underweight to the class I obese in both the physical and the mental component summary. Compared to the normal weight group, the class I obese had significantly higher scores in the mental component summary (MCS) (75.1 vs. 73.4, P<0.001). In contrast, the underweight group had the lowest scores in both the physical component summary (75.4vs. 77.5, P<0.001) and the mental component summary (71.8 vs. 73.4, P<0.001).

For eight dimensions, compared to the normal weight, the class I obese had significantly higher scores in the role-physical, general health, vitality, social functioning, role-emotional and mental health dimensions, whilst the class II obese had significantly lower scores in the physical functioning dimension with more than 3-point difference. The underweight had significantly lower scores in six dimensions (except physical functioning and role-emotional) and there was more than 3 points in the general health dimension. The overweight had the similar scores with normal weight in eight dimensions (Table 2). There was a small but discernible pattern of correlation between BMI and HRQL.

**Table 2:** The SF-36 subscale scores by BMI categories.

	Adjusted scores					Differences between BMI categories								
	Underweight	Normal weight	Overweight	Class I obese	Class II obese	Underweight *vs* Normal weight		Overweight *vs* Normal weight		Class I obese *vs* Normal weight		Class II obese *vs* Normal weight		*Overall P-value*
						D	*P-value*	D	*P-value*	D	*P-value*	D	*P-value*	
PF	88.1	88.8	88.9	88.4	85.4	-0.68	0.095	0.13	0.976	-0.36	0.602	**-3.43** * ^☂^	<0.001	<0.001
RP	75.5	77.7	78.7	79.6	77.6	**-2.14** *	0.005	1.03	0.255	***1*.*95*** *	*0*.*008*	-0.06	0.999	<0.001
BP	76.9	78.9	79.1	79.5	79.5	**-1.99** *	0.001	0.18	0.978	0.54	0.517	0.58	0.976	<0.001
GH	61.0	64.7	65.4	66.1	64.8	**-3.71** * ^☂^	<0.001	0.63	0.288	***1*.*33*** *	*0*.*004*	0.04	0.999	<0.001
VT	67.6	69.1	69.2	70.4	68.8	**-1.48** *	<0.001	0.15	0.982	***1*.*36*** *	*<0*.*001*	-0.19	0.999	<0.001
SF	81.0	82.5	83.3	83.8	85.2	**-1.45** *	<0.001	0.84	0.039	***1*.*30*** *	*0*.*001*	2.77	0.031	<0.001
RE	67.4	69.7	70.2	72.2	71.2	-2.30	0.016	0.43	0.949	***2*.*46*** *	*0*.*005*	1.51	0.930	<0.001
MH	71.1	72.4	72.9	74.0	74.7	**-1.30** *	0.001	0.52	0.328	***1*.*57*** *	*<0*.*001*	2.26	0.086	<0.001
PCS	75.4	77.5	78.0	78.4	76.8	**-2.13** *	<0.001	0.49	0.300	0.86	0.024	-0.72	0.892	<0.001
MCS	71.8	73.4	73.9	75.1	75.0	**-1.63** *	<0.001	0.49	0.416	***1*.*67*** *	*<0*.*001*	1.59	0.390	<0.001

(1) Abbreviations: PF, Physical Functioning; RP, Role Physical; BP, Bodily Pain; GH, General Health; VT, Vitality; SF, Social Functioning; RE, Role Emotional; MH, Mental Health. (2) BMI (kg/m^2^) categories: Underweight (<18.5); Normal weight (18.5–22.9); Overweight (23–24.9);Class I obese (25–29.9); Class II obese (≥30.0). D, difference mean value. (3) Scores of SF-36 was analyzed by standard least squares adjusted for gender, age, marital status, educational level, exercise habit, use of tobacco and alcohol, districts of residence and chronic conditions. (4) Analysis of Variance was used to get the *P* value. Dunnett test was applied to analyze the difference between groups.

*P<0.01.

(5)^**☂**^D ≥3 points is regarded as minimum clinically important difference (MCID).

### Correlation of BMI and HRQL Stratified by Status of Chronic Conditions

In the group of healthy, the BMI was associated with increasing scores in both the physical and the mental component summary from the underweight to the class I obese. Compared to the normal weight group, the class I obese had significantly higher scores in the mental component summary (MCS) (77.3 vs. 75.8, P = 0.004), and the underweight group had significantly lower scores in both the physical component summary (79.9 vs. 81.6, P<0.001) and the mental component summary (74.6 vs. 75.8, P = 0.009). In the group of chronic conditions, the SF-36 scores increased with increasing BMI from the underweight to the class I obese in the physical component summary, whilst from the underweight to the class II obese, it was associated with increasing scores in the mental component summary. The underweight had more than 3-point difference and significantly lower scores in both the physical component summary (67.9 vs. 71.8, P<0.001) and mental component summary (67.0 vs. 70.5, P<0.001). This counterintuitive association of BMI and HRQL was similar in the healthy and those with chronic conditions, but it was significant in the participants with chronic conditions.

Moreover, in the eight dimensions, compared to the normal weight group, the SF-36scores of the healthy were reduced by 3 points or more in the bodily pain dimension of the class II obese, and in the general health dimension of the underweight group. For subjects with chronic conditions, the SF-36 scores of the class II obese reduced more than 3 points in the physical functioning dimension and increased more than 3 points in the social functioning dimension. HRQL of the underweight group was reduced more than 3 points in most of the dimensions, with a maximum reduction of 5.43points (Table 3).

**Table 3:** The SF-36 subscale scores by BMI categories and status of chronic conditions.

	Adjusted scores					Differences between BMI categories								
	Underweight	Normal weight	Overweight	Class I obese	Class II obese	Underweight *vs* Normal weight		Overweight *vs* Normal weight		Class I obese *vs* Normal weight		Class II obese *vs* Normal weight		*P-value*
						D	*P-value*	D	*P-value*	D	*P-value*	D	*P-value*	
**Healthy**														
PF	90.7	91.1	91.4	90.7	88.2	-0.44	0.468	0.33	0.657	-0.34	0.780	-2.90	0.091	0.033
RP	80.9	82.4	83.3	84.3	82.2	-1.51	0.098	0.93	0.440	1.87	0.057	-0.20	1.000	0.003
BP	81.8	83.3	83.5	83.7	80.2	**-1.55** *	0.002	0.22	0.971	0.36	0.918	**-3.10** ^☂^	0.325	0.002
GH	66.3	69.7	70.4	70.2	67.8	**-3.39** * ^☂^	<0.001	0.71	0.323	0.52	0.782	-1.86	0.804	<0.001
VT	70.4	71.5	71.8	72.2	69.6	-0.99	0.063	0.44	0.676	0.83	0.262	-1.78	0.766	0.015
SF	83.7	84.7	85.4	85.8	84.8	-1.08	0.025	0.62	0.321	1.01	0.097	0.04	1.000	0.003
RE	72.4	74.1	74.7	76.6	72.3	-1.66	0.200	0.61	0.902	2.54	0.037	-1.83	0.978	0.016
MH	72.0	73.1	74.0	74.6	73.9	-1.11	0.019	0.84	0.084	***1*.*48*** *	*0*.*004*	0.75	0.985	<0.001
PCS	79.9	81.6	82.2	82.3	79.6	**-1.72** *	<0.001	0.55	0.289	0.60	0.395	-2.02	0.504	<0.001
MCS	74.6	75.8	76.5	77.3	75.1	**-1.21** *	0.009	0.63	0.303	***1*.*47*** *	*0*.*004*	-0.70	0.988	<0.001
**Chronic conditions**														
PF	83.1	84.6	84.0	83.9	80.7	-1.51	0.257	-0.62	0.719	-0.72	0.529	**-3.83** * ^☂^	0.008	0.016
RP	66.0	70.8	71.6	72.4	70.6	**-4.75** ^☂^	0.037	0.83	0.935	1.61	0.495	-0.23	1.000	0.029
BP	69.3	73.3	73.4	73.9	75.6	**-4.06** * ^☂^	<0.001	0.05	1.000	0.58	0.850	2.27	0.462	<0.001
GH	53.2	58.6	59.2	60.8	59.6	**-5.43** * ^☂^	<0.001	0.59	0.873	***2*.*17*** *	*0*.*005*	0.97	0.949	<0.001
VT	62.5	66.5	66.1	68.2	66.8	**-3.97** * ^☂^	<0.001	-0.38	0.961	1.63	0.031	0.23	0.999	<0.001
SF	75.8	79.0	80.3	80.7	83.2	**-3.16** * ^☂^	0.007	1.31	0.196	1.64	0.039	***4*.*15*** ^☂^	*0*.*020*	<0.001
RE	59.9	64.5	64.2	66.3	66.9	**-4.68** ^☂^	0.073	-0.36	0.998	1.76	0.487	2.42	0.866	0.042
MH	69.7	72.1	72.2	73.6	74.8	-2.45	0.020	0.03	1.000	1.50	0.025	2.63	0.146	<0.001
PCS	67.9	71.8	72.0	72.8	71.6	**-3.94** * ^☂^	<0.001	0.21	0.994	0.91	0.369	-0.21	0.999	<0.001
MCS	67.0	70.5	70.7	72.2	72.9	**-3.56** * ^☂^	<0.001	0.15	0.999	1.64	0.025	2.36	0.304	<0.001

(1) Abbreviations: PF, Physical Functioning; RP, Role Physical; BP, Bodily Pain; GH, General Health; VT, Vitality; SF, Social Functioning; RE, Role Emotional; MH, Mental Health. (2) BMI (kg/m^2^) categories: Underweight (<18.5); Normal weight (18.5–22.9); Overweight (23–24.9);Class I obese (25–29.9); Class II obese (≥30.0). D, difference mean value. (3) Scores of SF-36 was analyzed by standard least squares adjusted for gender, age, marital status, educational level, exercise habit, use of tobacco and alcohol, and districts of residence. (4) Analysis of Variance was used to get the *P* value. Dunnett test was applied to analyze the difference between groups.

* *P*<0.01.

(5)^**☂**^D ≥3 points is regarded as minimum clinically important difference (MCID).

## Discussion

The study evaluated the association between BMI and HRQL in Chinese adults. Potential confounders were adjusted including gender, age, marital status, educational level, exercise habit, use of tobacco and alcohol, districts of residence and chronic conditions. Overall, the class I obese had better HRQL in both the physical and the mental domains than the normal weight, especially in the mental well-being. The class II obese mainly had an adverse association in the physical functioning activities such as showering, dressing, and lifting heavy objects, but not all of the difference of SF-36 scores were significant in the physical and the mental dimensions. By contrast, the HRQL scores of the underweight were the lowest in both the physical and the mental component summary. This "obesity-HRQL paradox" phenomenon was more obvious in the participants with chronic conditions.

Generally considered overweight and obesity were negatively associated with worse outcome. Nevertheless, a new review of some research showed that overweight and moderately obese(mainly refers to the class I obese) elderly or patients who were overweight or obese lived longer and responded better to treatment, It's called the “obesity paradox”. Recent studies have shown the “obesity paradox” phenomenon still existed between BMI and HRQL. A cross-sectional study of 3,605 subjects conducted by López-García [20] to examine the correlation between body weight and HRQL in the population aged 60 and over in Spain found that, compared with normal weight subjects, obesity in fact correlated with higher HRQL on the SF-36 mental scales. Tsai [21] conducted a study to explore the impact of obesity in Taiwanese people, the result indicated that only the physical functioning dimension was significantly negatively associated with obesity, and was limited to class II obese (BMI≥30kg/m^2^). In a recent meta-analysis, Ul-Haq et al. [23] reported that different patterns were observed for the physical and mental HRQL. The physical HRQL significantly reduced in adults according to the degree of obesity. Compared to the normal weight the mental HRQL also reduced in adults with the most severe obesity, and the mild and moderate obesity had no difference in the mental HRQL, while the overweight significantly increased. Further evidence had been provided by Zhu [27] which intended to estimate the association between BMI and HRQL in middle-aged or older Chinese adults, and the observation showed that “obesity paradox” existed in middle-aged and older Chinese adults. Underweight subjects reported poorer HRQL in both the physical and the mental domain, while obese people had poorer physical function but a better mental health condition. Our results were consistent with these previous reports.

Our results also indicated that underweight group had the lowest HRQL scores in the physical and the mental domain, which were consistent with previous studies [26,41–43]. However, some studies emphasized the association between obesity and HRQL [10–13], and failed to include a comparative analysis of the underweight group relative to other body weight groups [44,45]. There was substantial percentage of underweight persons [22,46] in Asian countries, including China. In view of this, the neglected underweight persons need as much attention as obesity.

## Strengths and Limitations

Most evidence of the “obesity paradox” presented in previous studies had sparked lively discussions on how to explain this mechanism [47–49] and what have to do with the correlation between BMI and mortality [16,17,19,50]. The “obesity paradox” may be explained by early detection of diseases and initiation of treatments [51–54], greater metabolic reserves [17,47], and a mistaken definition of obesity based on the BMI [55]. Ul-Haq et al.[56] explored the “healthy obesity” by using data from a Scotland-wide survey by comparing the HRQL across the BMI category of people in the presence and absence of metabolic comorbidity, and addressed the question that there was a significant interaction with metabolic comorbidity (p = 0.007). The present study has extended the “obesity paradox” to HRQL outcomes in the Chinese adults. Combining with this study, it could be concluded that the class I obese group had the best quality of life with previous studies of HRQL as an independent predictor of death in patients [57–60]. Our results suggested that HRQL might be the intervening variable between obesity and prognosis. It means that obesity not only affects the prognosis directly but also influences the prognosis indirectly through HRQL. This notion might be a new perspective for the research.

Obese elderly were less likely to suffer from depressive symptoms than those of normal weight; this was confirmed in a study from Hong Kong in China [61]. Our results also supported obesity had better HRQL in the mental component summary. In China, the lifetime prevalence of major depressive disorder (MDD)was 3.5% [62] and the 12-month MDD prevalence was 2% [63]. As our results of “obesity paradox”, the research on body mass management of depressive disorder Chinese is very important.

Many chronic diseases are common in south Asians who have a lower BMI. For many Asian populations, additional trigger points for public health action were identified as 23 kg/m^2^ or higher, representing increased risk, and 27.5 kg/m^2^ or higher, as high risk. We conducted our research using the BMI classification endorsed by the WHO guidelines for the Asian Pacific population so that our results were useful for the situation in Asian. The validity of the BMI as measurement of obesity has been questioned in recent researches on the subject. Litwin [64] suggested that waist-to-height ratio (WHtR) had a stronger gradient of correlation with the incidence of CVD than other indexes (BMI, %BF, WC, WHR). A cross-sectional study by Romero-Corral [55] showed that the accuracy of the BMI in diagnosing obesity was limited, particularly for individuals in the intermediate BMI ranges, men, and older persons. In the intermediate BMI range (25–29.9 kg/m2), this measurement failed to distinguish between %BF and lean mass in either gender. Still, some studies had shown that the Pearson correlation coefficient between the BMI and percentage of body fat (%BF) was as high as 0.7–0.8 [65,66] in both males and females. A systematic review and meta-analysis of studies that have assessed the use of the BMI to detect body adiposity have shown that the commonly used BMI cutoff values in diagnosing male and female obesity have high specificity [67]. A longitudinal study of 13,155 patients with cardiovascular disease (CVD) conducted by the Department of Human Performance and Sport Sciences at Winston-Salem State University found that the risk for mortality from CVD correlated with BMI, and the researchers also observed a similar pattern for %BF [68]. Therefore, further researches are needed in order to determine the best method for quantifying adiposity.

In population studies, the BMI is usually calculated on the basis of self-reported body weight and height. The self-reporting of anthropometrics has been known to be biased resulting in the misclassification of BMI status [69]. However, Dekkers [70] suggested that self-reported BMI was sufficiently accurate to assess the prevalence of overweight/obesity in the middle-aged overweight working population. In this study, we collected self-reported weight and height from the participants and we did not attempt to determine whether self-reporting had any effect on accuracy.

## Conclusions

In this large population-based study, we found that the class II obese mainly had an adverse association on physical functioning, such as showering, dressing, and lifting heavy objects. The class I obese had the best HRQL in the physical domain and it also had the highest score in the mental domain. By contrast, the underweight group had the lowest HRQL scores in both the physical domain and the mental domain. This paradoxical association was significant in persons with chronic conditions.
